# A comparison of decisions to discharge committed psychiatric patients between treating physicians and district psychiatric committees: an outcome study

**DOI:** 10.1186/s13584-017-0178-8

**Published:** 2017-10-26

**Authors:** Daniel Argo, Igor Barash, Gadi Lubin, Moshe Z. Abramowitz

**Affiliations:** 1 Jerusalem Mental Health Center, Eitanim Psychiatric Hospital, Jerusalem, Israel; 2 Jerusalem Mental Health Center, Jerusalem, Israel; 3 Hebrew University-Hadassah School of Medicine, Jerusalem, Israel

**Keywords:** Involuntary hospitalization, Outcome, Quality improvement

## Abstract

**Background:**

The Israel Mental Health Act of 1991 stipulates a process for involuntary psychiatric hospitalization (IPH). A patient thus hospitalized may be discharged by either the treating psychiatrist (TP) or the district psychiatric committee (DPC). The decision rendered by the DPC is often at odds with the recommendation of the TP. Although much has been written about the ethical issues of restricting patients’ rights and limiting their freedom, far less attention has been devoted to the psychiatric, medical, and social outcome of legal patient discharge against the doctor’s recommendation.

**Methods:**

In our study we examined the outcomes of the decisions made by the DPC using readmission data, an internationally recognized indicator of the quality of hospital care, and compared them to the outcomes of patients discharged by the TP. All IPH discharges resulting from the DPC’s determination for the year 2013 (*N* = 972) were extracted from the Israel national register. We also collected all IPH discharges owing to the TP’s decision for 2013 (*N* = 5788). We defined “failure” as readmission in less than 30 days, involuntary civil readmission in less than 180 days, and involuntary readmission under court order in less than 1 year.

**Results:**

The rehospitalization pattern was compared in the two groups of patients discharged from their psychiatric hospitalization during 2013 (index discharges) and followed up individually for a year.

We found a statistically significant difference between the DPC and the TP group for each of the time frames, with the DPC group returning to IPH much more frequently than the TP group.

Using cross-sectional comparison with logistic regression adjusted for age, gender, diagnosis and length of hospitalization, we found the probability of a decision failure in the TP group was significantly less with an OR of 0.7 (95% CI .586–.863), representing a 30% adjusted decrease in the probability for failure in the TP group.

**Conclusions:**

The results we present show that the probability of decision “failure” (readmission) was found to be significantly higher in the DPC group than in the TP group. It is often assumed that IPH patients will fare better at home in their communities than in a protracted hospitalization. This is frequently the rationale for early discharge by the DPC (30.1 days vs. 75.9 DPC and TP groups, respectively). Our results demonstrate that this rationale may well be a faulty generalization.

## Background

The issue of psychiatric patients’ rights and freedom is basic in any discussion of the interfaces among law, society, and medicine. Many examples of the problematics involved in restricting individual freedoms during involuntary treatment and admission have been cited in the literature [1–5].

Israel first passed the Mental Health Act in 1955 [6], which gives the district psychiatrist the power to admit a mentally ill patient to a psychiatric ward against his/her will.

The original law reflected a somewhat paternalistic attitude, granting the medical/psychiatric profession considerable authority in the process, but a subsequent revision in 1991 [7] gave increased authority to commit a patient to a district psychiatric committee (DPC). In Israel, the DPC, which is made up of two psychiatrists and headed by an attorney at the level of a magistrate, decides whether to approve involuntary psychiatric hospitalization (IPH) and serves as an appellate committee for appeals lodged by patients. The IPH process is initiated by the district psychiatrist, independently of the hospital. Initially, the district psychiatrist has the authority to order seven days of involuntary IPH and to extend it for another seven days. Afterward, only the DPC can decide whether to prolong the IPH or to discharge the patient.

The Israel Mental Health Act requires “convincing evidence” (of psychiatric illness) to warrant IPH, not unlike the U.S. system in which a Texas trial court employed the standard of “clear, unequivocal and convincing” evidence deemed constitutionally adequate on appeal [8].

Appeals regarding orders for hospitalization, involuntary outpatient treatment, or discharge from hospitalization against the patient’s will are also decided upon by the DPC.

Subsequently, the Patient’s Rights Law enacted in 1996 [9] was designed to ensure the rights of all patients, including those of psychiatric patients. This led in 2004 to an amendment requiring the Ministry of Justice to provide legal representation to every committed patient [10]. Other countries, including Canada and the United Kingdom, have a similar system whereby medical and legal representatives decide on IPH [11, 12].

Whereas this law adds a layer of rights, the process engendered criticism owing to the development of long, drawn-out legal debates as opposed to brief to the point legal hearings regarding the situation of the committed patient [13, 14].

Both disciplines – medical and legal – have the patient’s best interests at heart, but the two try to achieve this goal in different ways based on their guiding principles. The psychiatrist views psychosis as interfering with an individual’s freedom and free will, whereas from the legal point of view the patient’s explicit will should be a major consideration, even if he/she is psychotic. In the event, the individual’s right to freedom of choice may override his medical interests.

In 2015, a total of 33.9% of all hospitalized psychiatric patients were either under a “hospitalization order” issued by the district psychiatrist or a court order (personal communication of data soon to be published by the Ministry of Health, Mental Health Services, Department of Information and Evaluation). This translates into 7742 patients a year hospitalized against their will, a steady increase in recent years (median increase of 0.8% per year, with increases in 9 out of 10 years between 2006 and 2015). With this number increasing and with the DPC review tribunals becoming more and more adversarial, the DPC has assumed an increasingly pivotal role in the medical/psychiatric outcome for psychiatric patients.

The decision rendered by the DPC is often at odds with the recommendation of the treating psychiatrist (TP). A hospitalized psychiatric patient can be discharged based simply on a note from the TP, but a decision to prolong the hospitalization order requires approval from the DPC.

Much has been written about both the ethical issues of restricting patients’ rights and limiting their freedom (1–5) and the arguments around the decision to discharge from hospitalization [15, 16]. Less has been written regarding the psychiatric, medical, and social outcomes of patient discharge against the doctors’ recommendation.

A limited study carried out in Israel did not lead to unequivocal conclusions [17]. Donisi et al. in an exhaustive recent literature review [18], which searched 734 articles, found that the legal status of the index admission was considered among the potential predictors in nine papers, but those papers only dealt with voluntarily admitted patients versus court-order admitted patients. In this same review, the type of discharge, whether escape from the hospital or discharge against medical advice, was also mentioned in only two papers, but there was no reference to cases of court-sanctioned discharge versus medical discharge.

A similar comprehensive literature review [19] found a dearth of research on the nature of the admission (voluntary, involuntary) correlated with readmission rates, and concluded that it is “difficult to draw conclusions from the results of these limited analyses.” The review does not cite any research on psychiatric readmissions and their association with medico-legal decisions to discharge committed patients.

Readmission rates are commonly used as indicators of the quality of hospital care [20, 21]. In a 10-year follow-up study, Rosca et al. showed that patients who were admitted involuntarily had a significantly greater number and longer durations of rehospitalizations than those who were admitted voluntarily [22]. We also subscribe to the view that the hospital readmission rate is a reliable quality indicator and valid for evaluating the outcome of the legal procedure that has been added onto the routine practice of discharge of IPH patients by the TP. In our study we examined the outcomes of the decisions made by the DPC using readmission data and compared them to the outcomes of patients discharged by the TPs.

## Methods

This outcome study is based on data from the Israeli National Psychiatric Hospitalization Registry of the Ministry of Health, which contains complete information on all psychiatric admissions in Israel since 1950 [23]. Approximately 22,000 hospitalizations are recorded annually. In order to analyze the outcomes for discharged patients, we extracted information on all the psychiatric discharges of IPH patients that resulted from the DPC’s determinations and all IPH discharges that were due to a TP’s decision for the year 2013 (*N* = 972 and *N* = 5788, respectively). In both extractions we included cases in which only a part of the total hospitalization was involuntary (i.e., the patient was initially hospitalized involuntarily, but after a period of treatment, he/she eventually became competent to sign a consent form).

Readmission rates were defined as the number of admissions for each time frame (within 30, 180, and 365 days) divided by the total number of cases. That is, when patients are discharged from the hospital, they are followed for one calendar year to check for readmissions. If any admission to the same or different hospital occurs during this time period, it is counted as a readmission.

Not included in the study were individuals above age 65 or below age 18 (23 such cases were removed from the DPC group; none from the TP group).

Also not included were patients with an ICD-10 “Z” diagnosis (“Z” codes represent reasons for encounters), ICD-10 F00-F09 diagnoses (dementia, delirium, etc.), ICD-10 F70-F79 diagnoses (mental retardation), and ICD-10 F80-F89 diagnoses (pervasive and specific developmental disorders). A total of 49 cases were removed from the DPC group and 907 from the TP group. The rationale for this exclusion was the intent to analyze the outcomes for individuals suffering from mental illness (i.e., schizophrenia, bipolar disorder, etc.), as opposed to a small number of those with situational crises, mental retardation, and similar diagnoses, which only infrequently come before the DPC and are treated somewhat differently.

We also excluded court-ordered hospitalizations (i.e., admissions for observation before trial for forensic purposes), which can only be rescinded by the court or by the DPC, so as to be able to compare the patient population in the two groups. A total of 422 and 644 cases were excluded from the DPC and TP groups, respectively.

Lastly, we excluded those who could not be rehospitalized owing to mortality; 7 patients were deceased in the DPC group and 72 in the TP group. The large number in the doctors’ group is an artifact: a hospitalized patient who dies (even when on leave) can be administratively discharged only by a doctor’s signature, causing an artificially large number to appear statistically.

In sum, we examined 4636 decisions (471 made by the DPC group and 4165 by the TPs), representing 3949 individual patients (443 and 3506 in the DPC and the TP groups, respectively) (see Table 1). Many of the patients were discharged and rehospitalized during the course of the follow-up year, 2013–2014.

**Table 1:** Exclusion and inclusion in the study

Step	Committee (DPC)	Doctors (TP)
Number of records received from Ministry of Health	972	5788
Number of records excluded by age criteria	23	0
Number of records excluded by diagnosis	49	907
Number of records excluded by procedural limitations	422	644
Number of records excluded by mortality	7	72
Final number of records included	471	4165
Final number of patients included	443	3506

### Statistical analysis

Statistical significance was set at *p* < 0.05 level. Chi-square tests and independent *t*-tests were performed to analyze the effect of separate categorical and continuous variables, respectively. Multivariate Cox regression analysis (adjusted for age, gender, diagnosis and length of hospitalization) was used to determine the risk of rehospitalization in each group. The results were presented as a Hazard ratio (HR) with a 95% confidence interval. The difference in the risk of rehospitalization between the DPC and TP groups was estimated using the Kaplan-Meier method.

A logistic regression adjusted for age, gender, diagnosis and length of hospitalization was used for comparison of the success vs. failure in each group (DPC vs TP). The results are shown as an odds ratio (OR) with 95% confidence interval.

## Results

Table 2 compares the demographics (age and gender), diagnoses, and length of the index hospitalization of the patients in the two groups; those who were discharged by the DPC and by the TPs, respectively. We found no differences in gender distribution and age of the participants or in the ICD-10 diagnoses of the included patients. Essentially the two groups studied were, statistically speaking, the same population.

**Table 2:** Demographic and index hospitalization history comparison

Measure	Committee (DPC)		Doctors (TP)		Difference	
	*N* (%)	*M* (SD)	*N* (%)	*M* (SD)		
Men	280 (63.2)		2223 (63.4)		χ^2^ = 0.106, *p* = .745	
Age		37.3 (12.3)		37.9 (12.3)	*t* = 1.079, *p* = .281	
Index hospitalization (days)		30.1 (71.2)		75.9 (315.3)	*t* = 7.775, *p* = .000	
Number of times previously hospitalized (average)		9.7 (15.4)		15.5 (29.5)	*t* = 6.830, *p* = .000	
Number of times previously hospitalized (median)		4		5		
Diagnoses	Committees (DPC)		Doctors (TP)		*χ* ^2^	*p*
F10-F19	4.5		5.0		4.481	.482
F20-F29	77.2		79.5			
F30-F39	12.2		11.0			
F40-F48	2.5		1.6			
F50-F59	0.0		0.1			
F60-F69	3.6		2.8			

F10-F19: Mental and behavioral disorders owing to psychoactive substance use, F20-F29: schizophrenia and schizotypal and delusional disorders, F30-F39: mood [affective] disorders, F40-F48: neurotic, stress-related, and somatoform disorders, F50-F59: behavioral syndromes associated with physiological disturbances and physical factors, F60-F69: disorders of adult personality and behavior

We did find a difference in the mean number of days of hospitalization in the index admission between the two groups: 30.1 days versus 75.9 for the DPC and TP groups, respectively. We believe that this is not an intrinsic difference in the two populations, but, rather, an inherent feature of the DPC’s decision process, which occurs almost exclusively when a TP is in disagreement with the committee (and the patient) and would recommend that the hospitalization be continued. The DPC’s decision to discharge a patient against the doctor’s recommendation, by definition, leads to shorter hospitalization durations.

We also found a statistically significant difference in the duration (days) of index hospitalization between the two groups, owing to the fact that some of the patients discharged by the doctors had multiple, protracted hospitalizations and suffered from a chronic course of the disorder. This explains why the median number of hospitalizations but not the average number of hospitalization days is similar in both groups, which skews the average number of days hospitalized upward. It is reasonable to assume that the doctors treating this group of chronic relapsing patients were cautious in making a decision to discharge, preferring to keep them hospitalized under order.

The rehospitalization pattern was compared in the two groups of discharged patients during 2013 (index discharges) and followed up individually for a year.

In Table 3 we show the differences in the frequency of rehospitalization between the groups within 30, 180, 365 days and above 365 days. We found a statistically significant difference between the DPC group and the TP group for each of the time frames, with the DPC group being rehospitalized much more frequently than the TP group.

**Table 3:** Differences in rates (%) of rehospitalization (cumulative)

Hospitalization type	Group	Within 30 days	Within 180 days	Within 365 days	HR/OR (95% CL)
Hospitalization (All)	Committees	20	43.7	55.8	
	Doctors	15.4	36.7	49.4	
	Difference	4.6	7	6.4	
	P	*χ* ^2^ = 6.617, *p* = .010	*χ* ^2^ = 9.046, *p* =** .003**	*χ* ^2^ = 6.887, *p* =** .009**	1.204 **(1.057–1.373)**
Involuntary hospitalization^a^	Committees	10.6	22.7	29.9	
(court and civil)	Doctors	6.1	15.2	21.4	
	Difference	4.5	7.5	8.5	
	P	*χ* ^2^ = 13.900, *p* = **.000**	*χ* ^2^ = 17.970, *p* = **.000**	*χ* ^2^ = 17.736, *p* = **.000**	1.225 **(1.022–1.469)**
Involuntary hospitalization^a^	Committees	1.5	3.2	4.5	
(court order)	Doctors	0.74	2.4	3.4	
	Difference	0.76	0.8	1.1	
	P	*χ* ^2^ = 2.865, *p* = .090	*χ* ^2^ = 1.23, *p* = .267	*χ* ^2^ = 1.445, *p* = .230	1.521 (.950–2.433)
“Success”/“Failure”^a^	Committees	20	32.1	33.3	
	Doctors	15.4	24.4	25.5	
	Difference	4.6	7.7	7.8	
	P	*χ* ^2^ = 6.617, *p* = .010	*χ* ^2^ = 13.021, *p* = **.000**	*χ* ^2^ = 13.493, *p* = **.000**	0.700 **(.586–.863)**

^a^The legal status of two rehospitalizations in the TP group was unclear and was not included.

Significance (*p* <0.005) and significant HR/OR are in bold

We then looked only at the IPH rehospitalizations, both civilian and by court order. This gives an indication of the clinical severity of the patients’ conditions upon readmission since they can only be hospitalized involuntarily if they are indeed deemed to be mentally ill and pose a threat to themselves or to others. Moreover, the long-term effects of rehospitalization include an erosion of trust between patient and doctor and restigmatization, among other negative repercussions.

Again, we found a statistically significant difference between the DPC and the TP group for each of the time frames, with the former returning to IPH much more frequently than the latter.

When examining only court-ordered readmissions for observation owing to the commission of a crime, we found a trend with the DPC group returning to IPH more frequently than the TP group; however, the total number of these court-order cases was too small to determine statistical significance.

We used a Cox regression analysis (adjusted for age, gender, diagnosis and length of hospitalization) to determine the risk of rehospitalization in each group. We found a significant difference in total readmissions and total involuntary readmissions (court and civil) with the DPC returning to IPH much more frequently than the TP group. There is a similar trend among court-ordered readmissions but with no statistical significance, due to the smaller number of cases (HR 1.521 (.950–2.433) *p* = 0.08).

**Fig. 1:**
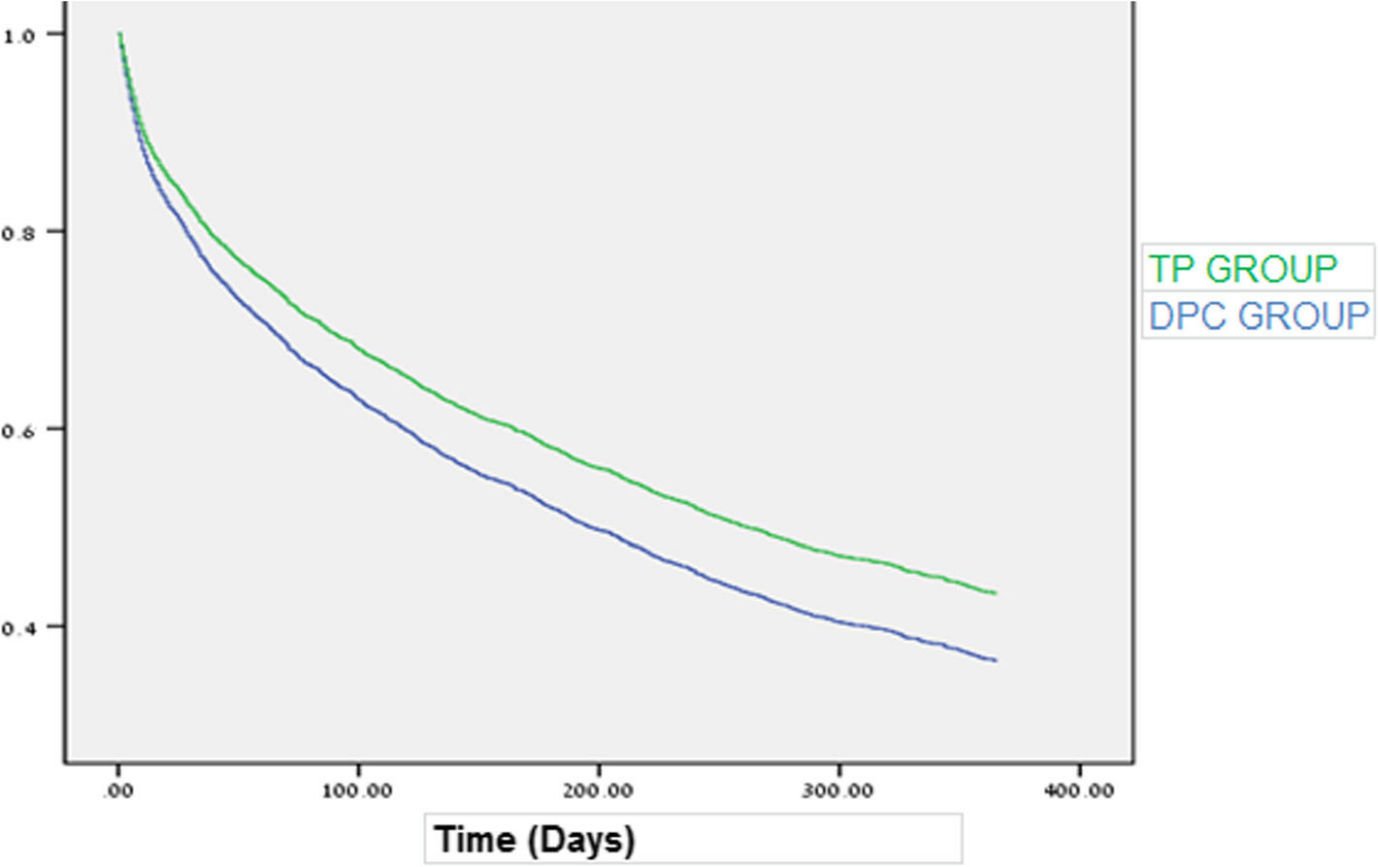
Adjusted rate of rehospitalization TP VS DPC (Kaplan-Meier survival curves)

Using Kaplan-Meier survival curves adjusted for age, gender, diagnosis and length of hospitalization, we compared the total rate of hospitalization in the DPC and TP groups (Fig. 1).

Among the widely used mental health care quality indicators, the OECD lists hospital readmission rates, noting that they “are widely used as proxies for relapse or complications following an inpatient stay for psychiatric and substance use disorders” [23]. In our study, we chose to define “failure” as readmission within 30 days or 180 days at any legal status and at 365 days by court order. No readmission during the calendar year after the index hospitalization or under a civilian order issued by the district psychiatrist after 180 days, or voluntary admission after 30 days post the index hospitalization was deemed a success (see Table 3).

We used logistic regression adjusted for age, gender, diagnosis and length of hospitalization for comparison of the success vs. failure in the TP and DPC groups. The probability of a failure in the TP group was significantly less, with an odds ratio of 0.7 (95% CI .586–.863) representing a 30% adjusted decrease in the chance for failure in the TP group.

Tables 4 and 5 show the success rates as function of ICD-10 diagnoses and gender among the two groups. The success rate of the TP group is higher than the DPC for all ICD-10 diagnoses and for both genders, and is statistically significant for psychotic disorders (*p* = .004), personality disorders (*p* = .006), and for men (*p* = .001). For other diagnoses, there is a clear trend but it is not statistically significant owing to the smaller sample size.

**Table 4:** Success rates (%) as function of ICD-10 diagnoses in the TP and DPC groups

Diagnoses	Committees	Doctors	*χ* ^2^	*p* Value
F10-F19	13 (61.9)	153 (74.1)	1.213	.271
F20-F29	241 (66.0)	2440 (73.8)	8.506	.***004***
F30-F39	44 (78.6)	364 (83.7)	1.132	.287
F40-F49	9 (81.8)	56 (91.8	1.059	.304
F50-F59	0 (0)	4 (80)	–	–
F60-F69	7 (41.2)	87 (73.7)	7.446	.006

F10-F19- Mental and behavioral disorders due to psychoactive substance use, F20-F29 - Schizophrenia, schizotypal, delusional, and other non-mood psychotic disorders, F30-F39 affective disorders, F40-F49 - Anxiety, dissociative, stress-related, somatoform and other nonpsychotic mental disorders, F50-F59- Behavioral syndromes associated with physiological disturbances and physical factors, F60-F69- Disorders of adult personality and behavior.

Significance (*p* <0.005) are in bold-italic

**Table 5:** Success rates (%) as function of gender in the TP and DPC groups

Gender	Committees	Doctors	*χ* ^2^	*p* Value
Men	189 (63.6)	1927 (72.5)	10.319	***.001***
Women	125 (71.8)	1177 (78.1)	3.504	.061

Significance (*p* <0.005) are in bold-italic

Zilber et al. [16] note that ultrashort IPH (i.e., less than 8 days duration) is an indicator for rehospitalization. Accordingly, we examined the success rates for the two groups for ultrashort index IPH (less than 8 days duration), short-term index IPH (between 9 and 30 days), and for above 30 days index hospitalization (see Table 6). We found that the TP had a significant success rate advantage (*p* = .003) over the DPC for the short-term IPH. The ultrashort and more than 30 day IPH show a trend favoring the TP group, but does not reach statistical significance. As previously noted, short periods of hospitalization have generally poorer prognoses; however, the longer the duration (and treatment) of the IPH, the better the prognosis, which perhaps explains the relatively favorable success rates in both groups for the more than 30-day IPH.

**Table 6:** Success rates (%) as a function of length of index hospitalization (days)

Length of index hospitalization	Doctors’ success rate (*N*)	Committees’ success rate (*N*)	*p* Value
Ultrashort hospitalization <8 days	73.4% (533)	67.7% (164)	*χ*2 = 2.2 *p*= .138
Short hospitalization 8–30 Days	73.5% (1006)	63.5% (129)	*χ*2 = 8.8 ***p*** **= .003**
Long hospitalization>30 days	75.6% (1565)	71.2% (74)	*χ*2 = 1.039 *p*= .308

Significance (*p* <0.005) are in bold

## Discussion

Although the impact of the legal process on the mentally ill individual as an intervening factor has been reviewed from the aspect of human rights and jurisprudence, it has not been sufficiently scrutinized from the perspective of patients’ well-being. We view the DPC, prescribed by law, as a new variable that affects the course of an illness or, at the very least, the readmission rate. Just as we would examine the outcome of the effect of a novel surgical procedure versus the traditional one, in this study, we endeavored to examine the outcome of DPC decisions in comparison with the long-standing tradition of hospital discharge on doctors’ orders.

It is difficult to find true quality indicators in mental health care. Many such are simply process indicators [24], which tell us more about the smooth running of the system and the application of regulations than whether patients actually benefit from their treatment. Other proposed indicators fail to capture meaningful aspects of the quality of care or are not readily amenable to the extraction of the needed data (specifically when not dealing with a controlled research study, i.e., subjective improvement based on rating scales). In our study, we propose the use of an internationally recognized outcome quality indicator [25] (e.g., “Hospital Readmissions for Psychiatric Patients in 30 days”) and have adapted it for the outcome of IPH (enlarging the scope to include 180- and 365-day time frames and including the legal status upon readmission). To achieve this goal, we use readily available administrative data with regard to admissions and legal status. We defined the “failure” of a decision to discharge an IPH patient as a return to hospitalization within a given time frame (30 days), return to hospitalization under IPH within 180 days, and a return to hospitalization under court order within 365 days (more weight given to this because of the social and therapeutic repercussions of a criminal offense on the individual).The absence of readmission or readmission not under these definitions was deemed a “successful” decision to discharge the individual from the index admission. We believe these definitions are clear and unequivocal, with ample face and content validity as a legitimate outcome quality indicator. To our knowledge, this is the first such attempt to compare DPC and TP decisions to discharge IPH patients.

The results we present show that the probability of decision “failure” (readmission) was found to be significantly higher in the DPC group than in the TP group. This was a consistent finding, though not always with a sufficiently large sample size to achieve statistical significance across all time frames, ICD-10diagnoses, and gender. The results were significant after cross-sectional comparison with a 30% decrease in the chance of failure in the TP group. Indeed, the trend toward increased rehospitalization for the DPC group was the same as that found in a 21-year study comprising 673 schizophrenia patients who left hospital against medical advice versus a control group of 1345 patients who were discharged on a physician’s recommendation [26].

Many times it is assumed that IPH patients will fare better at home in their community than in a long hospital stay. This is often the rationale for the early discharge by the DPC (30.1 days vs. 75.9 for DPC and TP groups, respectively). In our study we found that patients discharged by the DPC returned to hospitalization 36 days (mean number of days of hospitalization in the index admission) earlier than those discharged by the TP (with a median of 211 days until rehospitalization for the TP group and 175 days for the DPC group).

Moreover, as we have shown in our research, their more frequent involuntary readmissions under court orders result in longer, more protracted psychiatric hospitalizations [22]. (According to unpublished data we obtained from the Ministry of Health, the length of an average court-ordered hospitalization was 152 days compared to 64 days in voluntary hospitalization and 53 days under civil order). These results demonstrate that this rationale of faring better at home may be a faulty generalization and that in many cases the early discharge results in readmission and a longer hospital stay.

The patients’ rights aspect of the addition of the DPC to the IPH process cannot be depreciated. It is important for every individual to be assured that his/her rights are protected and his/her freedom valued no matter what illness has befallen him/her. Our patients’ families rely on the system to protect these rights, and this is what informs the foundation for trust in the system. Clearly, any new medical or surgical procedure with unfavorable outcomes in comparison with the traditional method would probably not be sanctioned for use. At the very least, it would seem important to inform the patient and his/her family that the readmission rate is higher when the treating physician’s opinion is overridden by the DPC.

We believe, in accordance with Chassin’s commentary in 2012 [27], that the present system involving legal oversight on the part of the DPC should be modified so as to improve the outcome measures. An interesting model has been proposed recently by Fistein et al. [28] to divide the legal framework that deals with involuntary hospitalization into two settings: one used to enact soft paternalism and the other to provide legal justification for detention for psychiatric treatment – both depending on the patient’s clinical situation.

A “soft paternalism” would justify limitations on liberty, for the benefit of the person being limited, provided that they are unable to make a choice that would be consistent with their own interests. This is many times an appropriate and perhaps a more practical approach the DPC could adopt, instead of a conservative approach requiring a specific standard of “proof” of major illness to qualify insanity requiring hospitalization.

Adapting such a therapeutic jurisprudence stance rather than an adversarial one might well enhance the DPC’s role and improve the rehospitalization rate. This approach might also promote increased awareness of the crucial importance of treatment consistency and the rehabilitation process [29, 30].

Lastly, systematic and periodic reviews of the quality indicators described above across different locations should provide for a better way to grade our patients’ well-being while still safeguarding their basic human rights.

## Conclusions

The results we present show that the probability of decision “failure” (readmission) was found to be significantly higher in the DPC group than) in the TP group. It is often assumed that IPH patients will fare better at home in their communities than in a protracted hospitalization. This is frequently the rationale for early discharge by the DPC (30.1 days vs. 75.9 DPC and TP groups, respectively). Our results demonstrate that this rationale may well be a faulty generalization. Adapting a therapeutic jurisprudence stance rather than an adversarial one might well enhance the DPC’s role and improve the rehospitalization rate. This approach might also promote increased awareness of the crucial importance of treatment consistency and the rehabilitation process.
